# Dirac points and the transition towards Weyl points in three-dimensional sonic crystals

**DOI:** 10.1038/s41377-020-00416-2

**Published:** 2020-12-22

**Authors:** Boyang Xie, Hui Liu, Hua Cheng, Zhengyou Liu, Jianguo Tian, Shuqi Chen

**Affiliations:** 1 The Key Laboratory of Weak Light Nonlinear Photonics, Ministry of Education, School of Physics, TEDA Institute of Applied Physics, and Renewable Energy Conversion and Storage Center, Nankai University, 300071 Tianjin, China; 2 The Key Laboratory of Artificial Micro- and Nanostructures of the Ministry of Education and School of Physics and Technology, Wuhan University, 430072 Wuhan, China; 3 The Collaborative Innovation Center of Extreme Optics, Shanxi University, 030006 Taiyuan, Shanxi China; 4 The Collaborative Innovation Center of Light Manipulations and Applications, Shandong Normal University, 250358 Jinan, China

**Keywords:** Physics, Photonic crystals

## Abstract

A four-fold-degenerate three-dimensional (3D) Dirac point, represents a degenerate pair of Weyl points carrying opposite chiralities. Moreover, 3D Dirac crystals have shown many exotic features different from those of Weyl crystals. How these features evolve from 3D Dirac to Weyl crystals is important in research on 3D topological matter. Here, we realized a pair of 3D acoustic Dirac points from band inversion in a hexagonal sonic crystal and observed the surface states and helical interface states connecting the Dirac points. Furthermore, each Dirac point can transition into a pair of Weyl points with the introduction of chiral hopping. The exotic features of the surface states and interface states are inherited by the resulting Weyl crystal. Our work may serve as an ideal platform for exploring exotic physical phenomena in 3D topological semimetals.

## Acoustic metamaterials: Exotic sonic surface states and their transition

The realization of exotic surface states in an acoustic metamaterial opens the door to new ways of manipulating sound waves. Some acoustic metamaterials behave like the sonic equivalents of topological insulators, which are insulators in their bulk but conduct electricity on their surfaces. Now, Shuqi Chen at Nankai University in Tianjin and co-workers have shown that the surfaces of a sonic crystal can conduct sound by supporting three-dimensional (3D) Dirac points – four-fold degenerate band crossings that possess topological charge. The researchers used 3D printing to build a sonic crystal from hexagonal unit cells, linked by tube structures that enable hopping between acoustic ‘atoms’. When chiral hopping is introduced, it allowed Dirac points to split into so-called Weyl points, enabling the transition of exotic surface states and interface states.

## Introduction

A three-dimensional (3D) Dirac point is a four-fold band crossing in 3D momentum space, away from which the energy band exhibits linear dispersion in all directions^1^. Recently, 3D Dirac points have been found in symmetry-protected crystals, such as Na_3_Bi^2^, Cd_3_As_2_^3,4^ and PtTe_2_^5^. As a fundamental topological band structure, a 3D Dirac point can transit into topological band gaps^6,7^, line nodes^8^ or Weyl points^9,10^. In particular, a 3D Dirac point can be treated as a degenerate pair of two Weyl points with opposite chiralities that can be separated in momentum space when their time-reversal symmetry or inversion symmetry is broken^1,9^. As a result, 3D Dirac semimetals share topological features with Weyl semimetals, such as Fermi arcs^11,12^ and chiral anomalies^13,14^. Moreover, 3D Dirac points can also exhibit exotic anomalous effects compared with Weyl points, such as spin-polarized surface states^15^, closed Fermi pockets^16,17^, and oscillating quantum spin Hall effect in quantum well structures^10,18^.

In the past few years, the concept of topological matter has inspired considerable research in photonics and acoustics^19–25^. Recently, acoustic topological semimetals have also been a focus of research as acoustic Weyl points^26–29^, spin-1 triple points^30^, and nodal lines^31,32^ have been discovered. Their exotic topological phenomena, including defect-tolerant transport^27^, topological negative refraction^29,30^ and one-way chiral zero modes^33^, have shown great promise for waveguide design and acoustic manipulation. In particular, 3D Dirac points, which can be distinguished into two classes, have recently aroused interest. Class I Dirac points are formed by band inversion. They lie on the generic momenta of an axis of rotation symmetry, always come in pairs and can be eliminated through merging and pairwise annihilation. The locations of the corresponding band crossings can be continuously tuned as a function of the Hamiltonian control parameters. Class II Dirac points are symmetry-enforced and unavoidable results of the nonsymmorphic space group of a material. They appear at high-symmetry points of the Brillouin zone. Their symmetries can support isotropic dispersions around such a Dirac point^34,35^. Recently, class II 3D Dirac points have been realized in classical systems using nonsymmorphic symmetry^34–36^. Meanwhile, class I 3D Dirac points created by band inversion have been discovered in photonics utilizing screw symmetries^37^, electromagnetic duality symmetry^15,38^ and hexagonal structures with $C_6$ symmetry^39^. However, topological helical surface states in class I 3D Dirac crystals have only appeared with electromagnetic duality symmetry. Since the design of topological states is more difficult without spin, class I 3D Dirac points and their topological states have not been realized in acoustic systems. Nevertheless, a class I Dirac point formed with fewer symmetry elements may provide an option for realizing strongly anisotropic dispersion. In addition, a class I Dirac sonic crystal, which mediates the phase diagram of a normal insulator phase and a topological insulator phase^40^ (weak topological insulator or topological crystalline insulator), may inherit topological surface states or hinge states^41^ from the topological insulator. These exotic features of class I Dirac sonic crystals may further inspire the design of topological sonic devices. Moreover, the transition from Dirac points to Weyl points has not yet been experimentally studied in either photonic or acoustic systems. For applications, it will be crucial to know how the surface states act during this transition.

In this paper, we report the theoretical and experimental realization of a pair of class I acoustic 3D Dirac points in a hexagonal sonic crystal and demonstrate how the exotic features of the surface states and interface states evolve in the transition towards Weyl points. The transition from two Dirac points to two pairs of Weyl points is realized by introducing chiral hopping into the Dirac sonic crystal. Correspondingly, the surface state dispersion evolves from connecting Dirac points to connecting Weyl points. Pseudospin-polarized helical states, which link the two Dirac points in momentum space, are created through particular interface design using sublattice pseudospin inversion. We find that the helical states can be inherited by the Weyl sonic crystal, while more exotic interface states can arise with the chirality inversion.

## Results

**Fig. 1:**
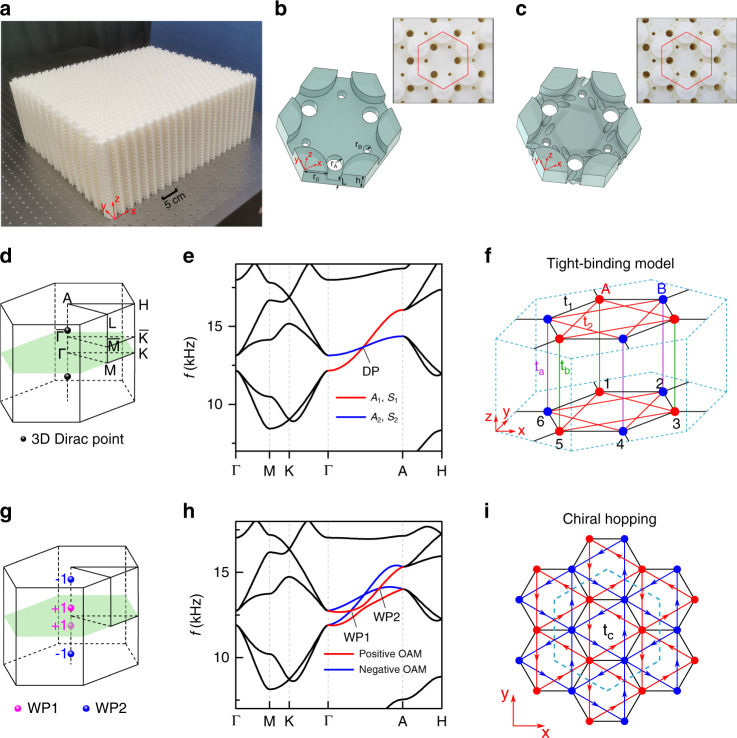
A 3D Dirac sonic crystal and its transition to a Weyl sonic crystal. **a** Photograph of the 3D Dirac sonic crystal. **b**, **c** Geometry of the unit cells for **b** the 3D Dirac sonic crystal and **c** the Weyl sonic crystal with additional chiral hopping tubes. The insets of **b** and **c** show top-view images of the 3D Dirac and Weyl sonic crystals. One unit cell is outlined with a red hexagon for each sonic crystal. **d** The Brillouin zone (BZ) of the 3D Dirac sonic crystal. **e** The band structure of the 3D Dirac sonic crystal where a Dirac point (DP) is located on ${\Gamma}A$. **f** Tight-binding model of the 3D Dirac sonic crystal, including nearest-neighbour hopping and next-nearest-neighbour hopping. **g** The BZ of the Weyl sonic crystal, where each 3D Dirac point splits into a pair of Weyl points, WP1 and WP2, with charges of +1 and −1, respectively. **h** The band structure of the Weyl sonic crystal. States with positive orbital angular momentum (OAM) and negative OAM are represented by red and blue lines, respectively. **i** Additional chiral interlayer coupling in the Weyl sonic crystal. The arrows represent the direction of positive phase hopping.

A Dirac sonic crystal was fabricated based on a layer-stacking strategy [Fig. 1a]. Schematic illustrations of the hexagonal unit cell for the 3D Dirac sonic crystal and the Weyl sonic crystal with additional chiral hopping tubes are shown in Fig. 1b, c, respectively. The samples were 3D printed with UV resin. The hexagonal unit cell has a lattice constant of *a* = 25.5 mm. The pillars are distributed at the corners of the unit cell on a perforated plate. The pillars have a height of 6 mm and a radius of *r*_0_ = 0.2223*a*. The fabrication error of the pillars is approximately 0.0003*a*, which may cause a 9 Hz gap. Such a narrow bandgap (~0.07% compared to the frequency) is difficult to probe in practice. The vertical tubes in the perforated plate constitute two interlaced triangular lattices that are symmetrically distributed around the pillars. The radii of the tubes are $r_{\mathrm{A}} = 2$ mm and $r_{\mathrm{B}} = 1$ mm, and the thickness of the plates is *L* = 4 mm. One feature of class I 3D Dirac points is that except for the high-symmetry point in the Brillouin zone (BZ), they are symmetrically located along the rotational axis ${\Gamma}A$, as shown in Fig. 1d. The double degeneracy along the ${\Gamma}A$ direction is protected by the *C*_3*v*_ symmetry, which has one two-dimensional (2D) representation. The band structure of the 3D Dirac sonic crystal is shown in Fig. 1e, where the tilted linear intersection point corresponds to the Dirac point. All numerical results were obtained through full-wave simulation using the finite-element method (FEM) software COMSOL Multiphysics. The velocity of the air is 343 m/s. The structure is considered to be acoustically rigid. A simplified tight-binding model of the 3D Dirac sonic crystal is shown in Fig. 1f. Here, we introduce the band folding mechanism by means of unequal next-nearest-neighbour (NNN) intralayer hopping. $t_2$ represents the NNN intralayer hopping within the unit cell. After band folding, the unit cell contains six “atoms” instead of two. As a result, the Weyl points at the *KH* and $K^{\prime}H^{\prime}$ lines of the original BZ fold to Dirac points at the ${\Gamma}A$ line of the new irreducible BZ. Compared with the band folding method in Ref. ^42^, our band folding method does not add any additional symmetry, and it forms a class I Dirac point. The details of the band folding mechanisms are presented in the Supplementary Information. The equal nearest-neighbour intralayer hopping $t_1$ is emulated by carefully choosing the pillar radius $r_0$ such that an accidental degeneracy can be formed by the dipole and quadrupole modes, which is similar to the 2D case^22^ (Supplementary Information and Fig. S2). The details of the tight-binding Hamiltonian and band dispersions are provided in the Supplementary Information. We obtain two two-fold degeneracies at ${\Gamma}A$, where the Hamiltonian in the basis $(A_1,S_1,A_2,S_2)^T$ is

1 $$H_0 = - t_2 + 2{\mathrm{cos}}\left( {k_zh} \right)\left( {\begin{array}{*{20}{c}} {t_a} & {} \\ {} & {t_b} \end{array}} \right) \otimes I_{2 \times 2}$$

Here, $A_1 = ( - 1,0,0,0,1,0)^T/\sqrt 2$, $S_1 = ( - 1,0,2,0, - 1,0)^T/\sqrt 6$, $A_2 = (0, - 1,0,0,0,1)^T/\sqrt 2$, and $S_2 = (0, - 1,0,2,0, - 1)^T/\sqrt 6$. *A* and *S* denote the asymmetric and symmetric modes, respectively, and 1 and 2 denote the A and B sublattice modes, respectively. Two accidental 3D Dirac points are formed at $(k_x,k_y,k_z) = (0,0, \pm k_{{\mathrm{DP}}})$, where $k_{{\mathrm{DP}}} = 0.5\pi /h$ if the on-site energies of the sublattices are the same. The tight-binding calculations along ${\Gamma}A$ are shown in Fig. S3.

Based on ${\mathbf{k}} \cdot {\mathbf{p}}$ perturbation theory, we can obtain the effective Hamiltonian $H_{{\mathrm{eff}}}({\bf{k}})$ near ${\Gamma}A$ in the basis $(A_1 + iS_1,A_2 - iS_2,A_1 - iS_1,A_2 + iS_2)^T$, where $A_1 \pm iS_1$ and $A_2 \pm iS_2$ have orbital angular momenta of $\pm 1$. Up to linear terms in ${\mathbf{k}}$, $H_{{\mathrm{eff}}}({\bf{k}})$ can be rewritten in block-diagonal form as

2 $$H_{{\mathrm{eff}}}({\mathbf{k}}) = \left( {\begin{array}{*{20}{c}} {h({\mathbf{k}})} & {} \\ {} & {h^ \ast ( - {\mathbf{k}})} \end{array}} \right)$$

where

3 $$h({\mathbf{k}}) = \left( {\begin{array}{*{20}{c}} { - t_2 + 2t_a{\mathrm{cos}}(k_zh)} & {A\left( {k_x - ik_y} \right)} \\ {A^ \ast \left( {k_x + ik_y} \right)} & { - t_2 + 2t_b{\mathrm{cos}}\left( {k_zh} \right)} \end{array}} \right)$$

and $A = - 3(\sqrt 3 - i)t_1a/4$. The Hamiltonian can be expanded with the sublattice pseudospin and orbital angular momentum to resemble the minimal $4 \times 4$ form for a Dirac semimetal^40^.

In the neighbourhood of the Dirac points, $h(\delta {\mathbf{k}})$ can be expressed in terms of Weyl points as follows:

4 $$h(\delta {\mathbf{k}}) = \left( {t_a + t_b} \right)\delta k_z\sigma _0 + {\sum} {\delta k_iv_{ij}\sigma _j,\,i,j \in \left\{ {x,y,z} \right\}}$$

To split the Weyl points in momentum space, an effective acoustic gauge flux is applied by adding interlayer chiral hopping in the structural design. The slanted connecting tubes have a radius of 0.7 mm. Their axes are 5.65 mm away from the central rotational axis and have an inclination angle of 65 degrees. As the symmetry of the sonic crystal is reduced from $C_{3v} \otimes I_z$ to $C_3$, each Dirac point is split into a pair of Weyl points on ${\Gamma}A$, as shown in Fig. 1g, h, where WP1 and WP2 are Weyl points with charges of +1 and −1, respectively. Chiral hopping with strength $t_c$ is considered in the tight-binding model [Fig. 1i], which introduces the following perturbation:

5 $$\delta H = \sqrt 3 t_c\sigma _z + t_c\delta k_zhI_{2 \times 2}$$

where $\vec \sigma$ denotes the Pauli matrices describing the orbital angular momentum. The degeneracy between the eigenstates $A_1 \pm iS_1$ and $A_2 \pm iS_2$ is now lifted. The $A_1 + iS_1$ and $A_2 - iS_2$ states are degenerate at $- k_{{\mathrm{WP1}}}$ and $k_{{\mathrm{WP2}}}$ with chiralities of +1 and −1, while the $A_1 - iS_1$ and $A_2 + iS_2$ states are degenerate at $- k_{{\mathrm{WP2}}}$ and $k_{{\mathrm{WP1}}}$ with chiralities of −1 and +1, where $0 \;<\; k_{{\mathrm{WP1}}} \;<\; k_{{\mathrm{DP}}} \;<\; k_{{\mathrm{WP2}}}$. The numerical simulation shows that the $A_1 + iS_1$ and $A_2 + iS_2$ states do not form a crossing, unlike in the tight-binding model. $A_1 + iS_1$ is further hybridized with $A_2 + iS_2$, forming a *d*-orbital-like bonding state $d_ + = A_1 - A_2 + i(S_1 - S_2)$ and a *p*-orbital-like antibonding state $p_ + = A_1 + A_2 + i(S_1 + S_2)$. Strictly speaking, the $A_1 - iS_1$ and $A_2 - iS_2$ bands do not form a crossing in the simulation either. In the tight-binding model shown in the Supplementary Information, the crossing points between $A_1{\,\mathrm{ + }}\,iS_1$ ($A_1 - iS_1$) and $A_{2} + iS_{2}$ ($A_2 - iS_2$) are not linear in directions perpendicular to *k*_*z*_, and they do not carry topological charge. Therefore, the $A_1\,{\mathrm{ + }}\,iS_1$ ($A_1 - iS_1$) and $A_2\,{\mathrm{ + }}\,iS_2$ ($A_2 - iS_2$) bands do not form Weyl points. The eigenstates for $k_z = 0.1\pi /h$ and $k_z = 0.4\pi /h$ are shown in the Supplementary Information and Supplementary Movie 1, showing band inversion and hybridization after $k_z$ crosses WP1. In addition to breaking the inversion symmetry with the chiral coupling, a time-reversal-breaking Zeeman field can also split the Dirac point. The Zeeman field can be a magnetic field in a photonic crystal or a circulating flow in an acoustic crystal^43^.

**Fig. 2:**
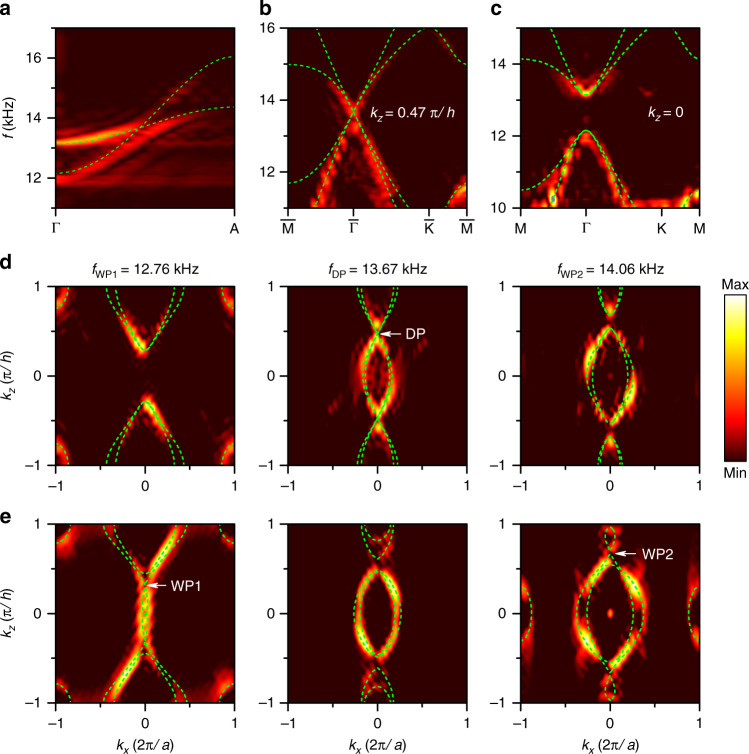
Experimentally mapped bulk band structure. **a** Band dispersion along the ${\Gamma}A$ direction. **b**, **c** Band dispersion in the reduced 2D planes at $k_z = 0.47\pi /h$ and $k_z = 0$. **d**, **e** Equifrequency contours with $k_y = 0$ for **d** the 3D Dirac sonic crystal and **e** the Weyl sonic crystal at frequencies of 12.76 kHz (WP1), 13.67 kHz (DP) and 14.06 kHz (WP2). The colour maps represent the strength of the states probed in the sonic crystal. The green dashed lines represent the calculated values obtained from full-wave simulations.

We probed the bulk states by Fourier transforming the experimentally measured pressure fields inside the sample, as shown in Fig. 2. A strongly tilted conical band crossing is clearly observed at $(k_x,k_y,k_z) = (0,0,0.47\pi /h)$ near 13.67 kHz along the ${\Gamma}A$ direction, as shown in Fig. 2a. A degeneracy is also found at the $\bar {\Gamma}$ point on the $k_z = 0.47\pi /h$ plane in Fig. 2b. For contrast, the band dispersion on the $k_z = 0$ plane is shown in Fig. 2c, where the four-fold degeneracy is lifted. The equifrequency contours (EFCs) of the bulk bands with $k_y = 0$ are shown in Fig. 2d. Two band pockets touch at the Dirac frequency and form linear crossings near the 3D Dirac points. For comparison, the EFCs for the Weyl sonic crystal are shown in Fig. 2e. The linear band crossings are formed at Weyl frequencies of 12.76 kHz and 14.06 kHz, while the degeneracies are lifted at a Dirac frequency of 13.67 kHz. The simulation results are well confirmed by the experimental results.

**Fig. 3:**
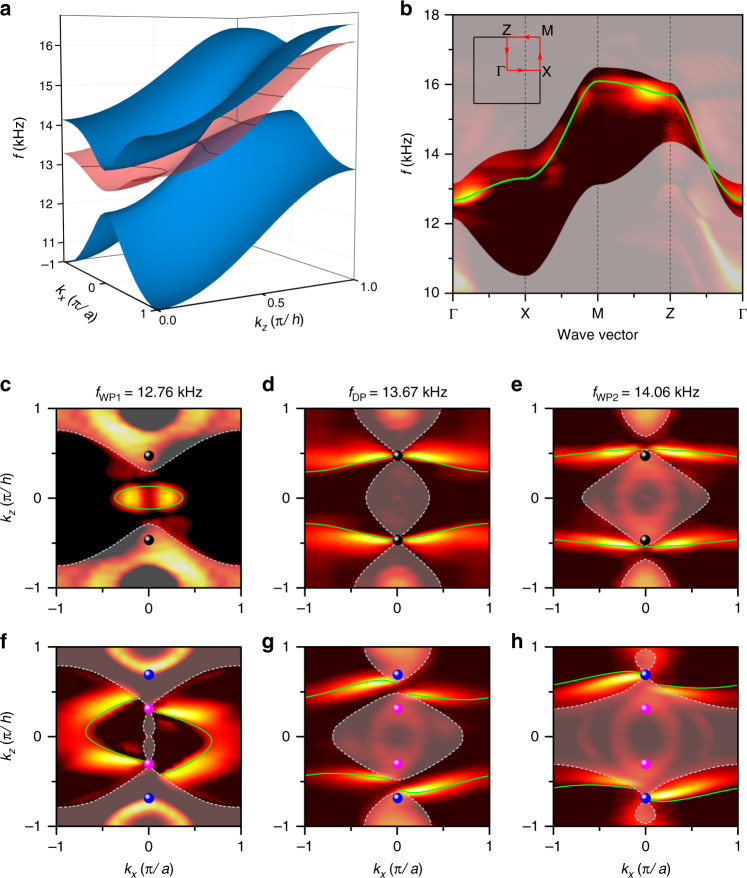
Surface states with a zigzag hard boundary. **a** 3D surface band structures of projected bulk states (blue) and surface states (red). **b** Surface band structure along the high-symmetry line of the surface Brillouin zone. **c**–**h** Equifrequency contours of the surface state for the **c**–**e** Dirac and **f**–**h** Weyl sonic crystals at frequencies of 12.76 kHz (WP1), 13.67 kHz (DP) and 14.06 kHz (WP2). The green lines represent the calculated surface states, while the shaded areas represent the calculated projected bulk bands. The black spheres represent the projections of the Dirac points. The pink and blue spheres represent the projections of the Weyl points.

While a Weyl semimetal possesses surface arc states on the surface as a manifestation of the topological singularities in the bulk band structure, a Dirac semimetal, regarded as a merger of two compensated Weyl points, can form similar surface arcs. The surface band dispersion for a zigzag rigid surface (normal to the $y$ direction) is shown in Fig. 3a, where a surface band appears between the projections of the bulk bands. To measure the surface states, we placed a sound source close to the centre of the surface for excitation. By Fourier transforming the measured field, the band structure along the high-symmetry line of the surface BZ was obtained, as shown in Fig. 3b. The EFCs at the Dirac frequency and Weyl frequencies are shown in Fig. 3c–e for the Dirac sonic crystal. The surface arc starts from the projection of the Dirac point and links the equivalent projected Dirac points, crossing the nearby BZ. Away from the Dirac frequency, these surface states do not connect the projections of the bulk bands because they are not topologically protected^44^. As the frequency decreases, the surface arcs of the $k_z\, >\, 0$ and $k_z\, <\, 0$ parts intersect at $k_z = 0$ and then transform into a closed pocket. When the Dirac points split into Weyl point pairs, topological surface states are observed linking the Weyl points with opposite chiralities, as shown in Fig. 3f–h. When the working frequency is not located at the Weyl frequency, the surface arc will connect the projections of bulk bands enclosing the Weyl points. The surface arcs of the Dirac sonic crystal can be viewed as surface arcs linking the paired Weyl points, consistent with the results for the Weyl sonic crystal. Additionally, the pressure field distributions at the surfaces of the Dirac and Weyl sonic crystals share the same vortex feature (see Fig. S6 in the Supplementary Information). The design of the surface shape will greatly affect the surface states. The surface band dispersions with a flat boundary are shown in Figs. S7–S9 in the Supplementary Information, where the surface states connect different Dirac points instead of self-connecting at the Dirac frequency. The Weyl sonic crystal can also support leaky surface states with a zigzag open boundary (Supplementary Information and Fig. S10).

**Fig. 4:**
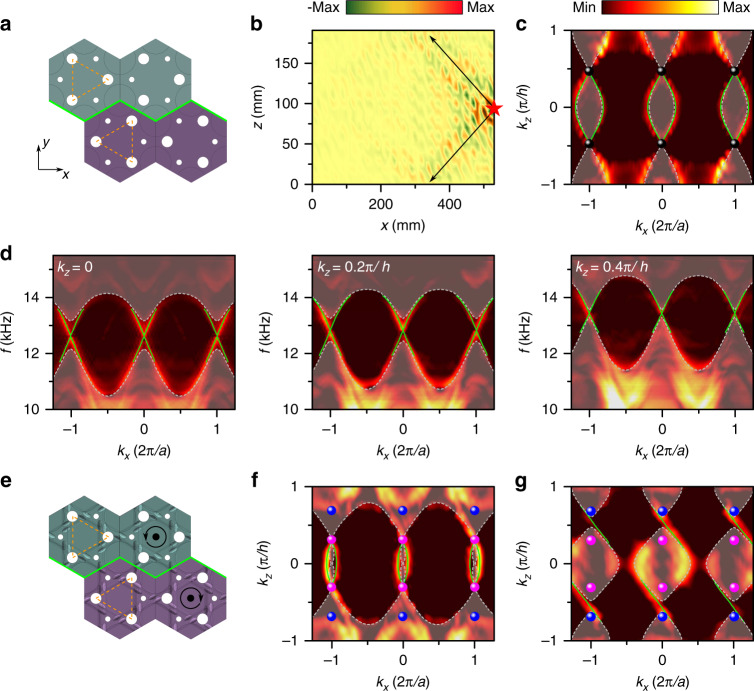
Observation of helical interface states in the 3D Dirac sonic crystal. **a** Schematic of the interface formed by swapping the large and small tubes. **b** Measured real-space interface wave propagation excited by a point-like source at the Dirac frequency. The position of the sound source is indicated by a red star. **c** Fourier-transformed equifrequency contours for the data in **b**. The black spheres represent the projections of the Dirac points. **d** Interface band dispersion for $k_z = 0$, 0.2$\pi /h$ and 0.4$\pi /h$. The green solid lines represent the simulated interface states. The shaded areas represent the calculated projected bulk bands. **e** Schematic of the interface formed by swapping the large and small tubes and inverting the chirality of slanted tubes of the Weyl sonic crystal. **f**, **g** Equifrequency contours at **f** WP1 frequency and at **g** Dirac frequency.

In electronic or photonic systems, double helical surface arc states are featured as a topological signature of Dirac points. Analogously to the quantum spin Hall effect, a Dirac sonic crystal can generate helical surface arcs if band inversion is introduced. Such a system can be effectively considered to exhibit the 2D quantum spin Hall effect for each $k_z$ such that $|k_z| \;<\; k_{{\mathrm{DP}}}$, which is characterized by a nontrivial $Z_2$ number^39^. Helical states that have opposite group velocities and angular momenta in certain $k_z$ planes are distinct features of a nontrivial *Z*_2_. In addition, the helical surface states show the nontrivial *Z*_2_ charge carried by the Dirac point^45^. Helical surface states not only can manifest interesting topological characteristics in theory but are also important in applications because the linear part of their dispersion enables transport with better efficiency and robustness. To realize pseudospin-polarized surface states, a zigzag interface was built by switching the positions of the large tubes and small tubes in one domain, as shown in Fig. 4a. In this domain, the energy of the $A_1 \pm iS_1$ states is exchanged with that of the $A_2 \pm iS_2$ states because the hopping terms $t_a$ and $t_b$ are swapped in Eq. (1), which can cause pseudospin inversion. This pseudospin inversion also gives rise to opposite pseudospin Chern numbers for the two different domains, leading to topological surface states. To measure the interface states, a point-like source was placed at the edge of the interface. The spatial field in Fig. 4b shows the propagating acoustic waves measured at the Dirac frequency, illustrating the up- and left-ward and the down- and left-ward branches of the surface waves. In the EFCs of the interface states [Fig. 4c], two branches of gapless interface modes appear at $|k_z| < k_{{\mathrm{DP}}}$ between the Dirac points, with one moving along $+ x$ and the other moving along $- x$. Although the interface modes are very close to the projection of the bulk band at the Dirac frequency, they contain much higher amplitudes with respect to the bulk states. The interface band dispersions for $k_z = 0$, $0.2\pi /h$, and $0.4\pi /h$ are shown in Fig. 4d. For these $k_z$ planes, a pair of interface states connecting the upper and lower bulk states forms a linear cone spectrum in the gap area.

The interface states of the Weyl sonic crystal become more complicated. The interface created through both sublattice pseudospin and chirality inversion is shown in Fig. 4e. For $0 \;<\; |k_z| \;<\; k_{{\mathrm{WP1}}}$, when the pseudospin is inverted, pseudospin-polarized interface states emerge in the EFCs at a frequency of $f_{{\mathrm{WP1}}}$ or lower, as shown in Fig. 4f. These pseudospin-polarized states are analogous to the interface states of the 3D Dirac sonic crystal shown in Fig. 4c. For the states on ${\Gamma}A$ with $k_{{\mathrm{WP1}}} \;<\; |k_z| \;<\; k_{{\mathrm{WP2}}}$, the upper (lower) bands share the same angular momentum depending on the chirality of the sonic crystal. With chirality inversion, gapless one-way interface states can also appear in Fig. 4g at 13.67 kHz between the Weyl frequencies. In this case, the difference in Chern number between the two domains is $\pm 2$ within the range of $k_{{\mathrm{WP1}}} \;<\; |k_z| \;<\; k_{{\mathrm{WP2}}}$. The one-way interface states and the pseudospin-polarized states belong to two different regions in wave vector space. The appearance of pseudospin-polarized states does not affect the one-way interface states. For comparison, the cases in which an interface is created only through pseudospin inversion or only through chirality inversion are shown in Fig. S11 in the Supplementary Information. The pseudospin-polarized interface states and the chiral interface states correspond to different regions in momentum space and different frequencies, which may further inspire the design of topological devices using both kinds of interface states.

## Discussion

In conclusion, we designed a class I Dirac sonic crystal in which type II Dirac points are formed on the rotation axis via accidental degeneracy. A class I Dirac point formed with fewer symmetry elements can provide an option for realizing strongly anisotropic dispersion. It can also provide ideal conditions to study the transition from Dirac points towards Weyl points. By introducing chirality into the sonic crystal, we split the two Dirac points into two pairs of Weyl points in momentum space, as clearly observed in the EFCs of the bulk bands. We observed that the surface states at a zigzag boundary evolve with the surface arcs from connecting Dirac points to connecting Weyl points. By creating an interface through band inversion, helical pseudospin-polarized interface states were also observed in both the Dirac and Weyl sonic crystals. The helical states in class I Dirac semimetals related to weak topological insulators can be topologically protected. An acoustic class I Dirac semimetal can surely provide a highly directional pseudospin-polarized surface, which may offer advantages over class II Dirac semimetals in some applications that require directional transport. The demonstrated sonic crystal system provides a platform for studying the transition between 3D Dirac points and Weyl points and may further inspire the design of weak topological insulators, the realization of acoustic hinge states, and the design of other 3D topological devices.

## Materials and methods

### Experimental measurements

A loudspeaker (diameter = 5.7 mm) was used as the sound source in the experimental measurements. The sound source was driven by a broadband pulse. For acoustic field measurements, an omnidirectional microphone (Panasonic WM-G10D, diameter = 4 mm, height = 1 mm) attached to the tip of a stainless-steel tube (diameter = 2.5 mm) was inserted into the sample through the space between the plates. Another microphone (B&K Type 4961) was fixed in place to serve as a phase reference. The acoustic signals were analysed using a multi-analyser system (B&K Type 3560B), with which both the wave amplitude and phase were extracted. Scanning was performed with a stage that could move in three directions. The bulk and surface dispersions of the acoustic system were obtained by Fourier transforming the measured fields.

## Supplementary information

Supplementary Information

Supplementary Movie 1
