# Supplementary material for: Dirac points and the transition towards Weyl points in three-dimensional sonic crystals
Source: Light Sci Appl. 2020 Dec 22;9:201. doi: 10.1038/s41377-020-00416-2 (PMC7755923; doi:10.1038/s41377-020-00416-2)
Supplement: Supplementary file 1 — Supplementary Information [file 41377_2020_416_MOESM1_ESM.doc]

Supplementary Information for

**“Dirac points and the transition towards Weyl points in**

**three-dimensional sonic crystals”**

Boyang Xie^1^, Hui Liu^1^, Hua Cheng^1,*^, Zhengyou Liu^2^ , Jianguo Tian^1^, and Shuqi Chen^1,3,4,*^

*^1^The Key Laboratory of Weak Light Nonlinear Photonics, Ministry of Education, School of Physics, TEDA Institute of Applied Physics, and Renewable Energy Conversion and Storage Center, Nankai University, Tianjin 300071, China*

*^2^The Key Laboratory of Artificial Micro- and Nanostructures of the Ministry of Education and School of Physics and Technology, Wuhan University, Wuhan 430072, China*

*^3^The Collaborative Innovation Center of Extreme Optics, Shanxi University, Taiyuan, Shanxi 030006, China*

*^4^The Collaborative Innovation Center of Light Manipulations and Applications, Shandong Normal University, Jinan 250358, China*

** e-mail: hcheng@nankai.edu.cn; schen@nankai.edu.cn*

1. **Band folding mechanisms**

We introduce band folding mechanisms in a $\sqrt{3}\times\sqrt{3}$ supercell. Fig. S1a shows the unit cell (blue) and supercell (red) of the Weyl crystal. Fig. S1b shows the corresponding Brillouin zone (BZ) BZ1 (blue) and BZ2 (red). The bulk dispersion follows the mapping relations^1^, which is shown in Fig. S1c that

$\overline{\Gamma_{0}}+\overline{K_{0}}+\overline{K'_{0}}|_{BZ1}=\bar{\Gamma}|_{BZ2}$, (or $\Gamma_{0}A_{0}+K_{0}H_{0}+K'_{0}H'_{0}|_{BZ1}=\Gamma A|_{BZ2}$)

$\Gamma_{0}K_{0}+K_{0}K+K'_{0}K|_{BZ1}=\Gamma K|_{BZ2}$.

By removing the pillar in the middle of the supercell, the supercell is reduced to the unit cell of Dirac crystal. The new unit cell has less rotational axis, thus the degeneracy along $\Gamma K$ direction is lifted due to symmetry breaking.

1. **Accidental degeneracy by structural design**

Generally, the two-fold degenerate states at $\Gamma A$ are dipole or quadrupole modes, whose eigenfrequencies depend on the radius of pillars $r_{0}$. Accidental degeneracy is formed at the critical point of $r_{0}=0.2223a$ with $\mathbf{k}=(0,0,0.47\pi/h)$ as shown in Fig. S2a. The pressure field distributions of the dipole and quadrupole modes are shown in Figs. S2b and S2c.

1. **Tight-binding calculations**

We consider a $6\times6$ Hamiltonian that describes the site-site hopping, as there are six “atoms” in a unit cell. We introduce a tight-binding model that includes inter-atomic hopping with nearest neighbours and next-nearest neighbours

where $c$ and $c^{\dagger}$ are the annihilation and creation operators on the sublattice sites, the subscripts $(i,j)$ represent the lattice sites, and $k$ represents the number of layers. $t_{1}$ represents the nearest-neighbour (NN) intralayer hopping, $t_{a}$ and $t_{b}$ represent the interlayer hopping for sublattice A and B. The last term represents the chiral interlayer hopping with strength $t_{c}$, where $v_{ij,k}=\left[(2/\sqrt{3})(\hat{\mathbf{e}}_{i,k}\times\hat{\mathbf{e}}_{j,k})_{z}+1\right]/2$. When $t_{a}\neq t_{b}$, Xiao *et al.* discovered that Weyl point can exist in high symmetry *KH* and $K'H'$ lines of the BZ^1^. We note that the Weyl degeneracy will be lifted when the NN intralayer hopping are not all equalled. To obtain a fourfold 3D Dirac point, here we introduce the band folding mechanism by using unequalled next-nearest-neighbour (NNN) intralayer hopping. $t_{2}$ represents the NNN intralayer hopping within the unit cell. The NNN intralayer hopping between the unit cells are insignificant so we neglect them in later analyses. By solving the tight-binding model, we obtain two two-fold degeneracy at $\Gamma A$ where the Hamiltonian is

$$H_{0}=-t_{2}+2\cos(k_{z}h)\left(\begin{matrix} t_{a} & \\ & t_{b} \end{matrix}\right)\otimes I_{2\times2}$$

in the basis $(A_{1},S_{1},A_{2},S_{2})^{T}$ in which $A_{1}=(-1,0,0,0,1,0)^{T}/\sqrt{2}$, $S_{1}=(-1,0,2,0,-1,0)^{T}/\sqrt{6}$, $A_{2}=(0,-1,0,0,0,1)^{T}/\sqrt{2}$, and $S_{2}=(0,-1,0,2,0,-1)^{T}/\sqrt{6}$. $A$ and $S$ denote the asymmetric and symmetric modes, respectively, and 1 and 2 denote the A and B sublattice modes, respectively. The band dispersion indicates that the 3D Dirac points can be type II when $t_{a}t_{b}>0$, or type III (critically tilted) when $t_{a}t_{b}=0$. Each Dirac point can transit towards a pair of Weyl points in $\Gamma A$ by introducing chiral hopping. The details are demonstrated in the main text.

We calculate the band dispersion along $\Gamma A$ through tight-binding model, as shown in Fig. S3. The hopping terms are $t_{1}=-1$, $t_{2}=-0.1$, $t_{a}=-0.6$, $t_{b}=-0.25$, $t_{c}=0$ for Dirac sonic crystal (Fig. S3a) and $t_{c}=-0.15$ for Weyl sonic crystal (Fig. S3b). The $A_{1}+iS_{1}$ and $A_{2}+iS_{2}$ bands form crossing in the tight-binding model instead of hybridizing in full-wave simulation. We also calculated the reduced $k_{z}$ planes at band crossing points in Figs. S3c-S3f. The band crossing is linear in all directions only between the $A_{1}+iS_{1}$ ($A_{1}-iS_{1}$) and $A_{2}-iS_{2}$ ($A_{2}+iS_{2}$). The other crossings are not Weyl points, as they are not linear in directions perpendicular to $k_{z}$.

Based on the tight-binding model, we calculated the topological charge of Dirac point and Weyl points through the evolution of the Berry phase over a sphere enclosing the crossing point^2^, as a function of polar angle *θ* in Fig. S4. The gapless spectra indicate a nontrivial monopole charge of $Z_{2}=1$ for Dirac point, and Chern number +1 or -1 for WP1 or WP2. In contrast, the topological charge around the crossing between $A_{1}+iS_{1}$ and $A_{2}+iS_{2}$ in Weyl crystal is trivial, indicating that the crossing point is not Weyl point.

1. **Eigenstates along rotational axis**

The eigenstates along $\Gamma A$ in Dirac sonic crystal are doubly degenerated. When the chiral hopping is introduced in the sonic crystal, the degenerated states are lifted into separate states of different angular momentums. The pressure fields at $k_{z}=0.1\pi/h$ and $k_{z}=0.4\pi/h$ are shown in Fig. S5 and Supplemental movie 1. For $k_{z}=0.1\pi/h$ between the $\Gamma$ point and WP1, the eigenstates from the second band to the fifth band are $A_{1}+iS_{1}$, $A_{1}-iS_{1}$, $A_{2}+iS_{2}$ and $A_{2}-iS_{2}$, which form the basis in our theory model. For $k_{z}=0.4\pi/h$ between WP1 and WP2, band inversion takes place after $k_{z}$ moves across WP1. $A_{1}-iS_{1}$ moves to the higher band and $A_{2}+iS_{2}$ moves to the lower band. $A_{2}+iS_{2}$ is further hybridized with $A_{1}+iS_{1}$ forming *d*-orbital-like $d_{+}=A_{1}-A_{2}+i(S_{1}-S_{2})$ and *p*-orbital-like $p_{+}=A_{1}+A_{2}+i(S_{1}+S_{2})$ states, therefore the $A_{1}+iS_{1}$ and $A_{2}+iS_{2}$ bands do not form crossing in band dispersions as shown in Fig. 1k.

1. **Vortex in surface states and interface states**

The field distribution of surface states at $(k_{x},k_{z})=(0.3\pi/a,0)$ is shown in Fig. S6, where vortex features appear in the waveguide area. Here the time-reversal symmetry at $k_{z}=0$ has eliminated the out-of-plane Poynting vector. The pressure amplitude vanishes at the vortex centre. The Poynting vector near the vortex centre clearly shows the vortex and its chirality. For surface states with zigzag boundary, a vortex emerges at the centre of each unit cell near the boundary, as shown in Figs. S6a and S6c for Dirac and Weyl sonic crystal. For zigzag interface formed though pseudospin inversion, the unit cell contains two vortices of opposite chiralities, as shown in Figs. S6b and S6d for Dirac and Weyl sonic crystal. As a feature of surface states for both Dirac and Weyl sonic crystal, the vortex chirality is dependent on the propagating direction of the surface states. This feature can be used to selectively stimulate surface wave propagation towards different directions.

1. **Surface states with flat boundary**

The surface band dispersion of Dirac sonic crystal with flat boundary is shown in Fig. S7. The 3D band structure on surface XZ is shown in Fig. S7a. The band structure along high symmetry line of surface BZ is shown in Fig. S7b, where eigenfrequencies around X, M and Z points of the surface BZ are much lower than the zigzag boundary case. The surface bands form a closed pocket at 12.0 kHz and 12.4 kHz as shown in Fig. S7c. The pocket expands and forms accidental linear crossing at 12.76 kHz. The crossing is further gapped by the bulk bands emerging around the $\Gamma$ point of the surface BZ. The surface band encloses one of the bulk bands at higher frequencies including the Dirac frequency. At Dirac frequency of 13.67 kHz, the surface states connect different Dirac points instead of self-connecting.

In the Weyl sonic crystal with flat boundary, the surface states only appear around the $\Gamma Z$ line of the surface BZ, while the surface states can appear in various area in the surface BZ, as shown in Figs. S8a and S8b. The band dispersion at $k_{z}=0.5\pi/h$ between the paired Weyl points are shown in Figs. S8c and S8d. Compared with surface bands at flat boundary, the trajectory of surface bands at zigzag boundary crosses the XM line ($k_{x}=\pm\pi/a$) before disappearing in the projection of bulk bands. Equifrequency contours of the surface states with flat boundary are shown in Fig S9, in which the surface states only exist between the two Weyl frequencies. The flat hard boundary for Weyl sonic crystal is similar to the interface with inversed chirality, because the flat boundary can be viewed as a mirror plane which reflects the chirality into the opposite direction.

1. **Surface states with open boundary**

The Dirac sonic crystal cannot support Fermi arcs at the interface with air, while the Weyl sonic crystal can support leaky Fermi arcs within the range of at zigzag interface. The simulation results are shown in Fig. S10. The Fermi arcs are leaky inside the acoustic cone of the airborne sound $f>c_{0}k/(2\pi)$.

1. **Interface dependent states of Weyl sonic crystal**

Fig. S11 shows the interface states when the interface is created by only pseudospin (Fig. S11a) or chirality inversion (Fig. S11b). The pseudospin-polarized states at $f_{\text{WP1}}$ emerge in Fig. S11c but not in Fig. S11d. For chirality inversion, the gapless one-way interface states appear in Fig. S11f but not in Fig. S11e at 13.67 kHz between the Weyl frequencies.

**References:**

1 Liu Y. Z. *et al*. Pseudospins and Topological Effects of Phonons in a Kekulé Lattice. *Physical Review Letters* **119**, 255901 (2017).

2 Soluyanov A. A. *et al*. Type-II Weyl semimetals. *Nature* **527**, 495-498 (2015)


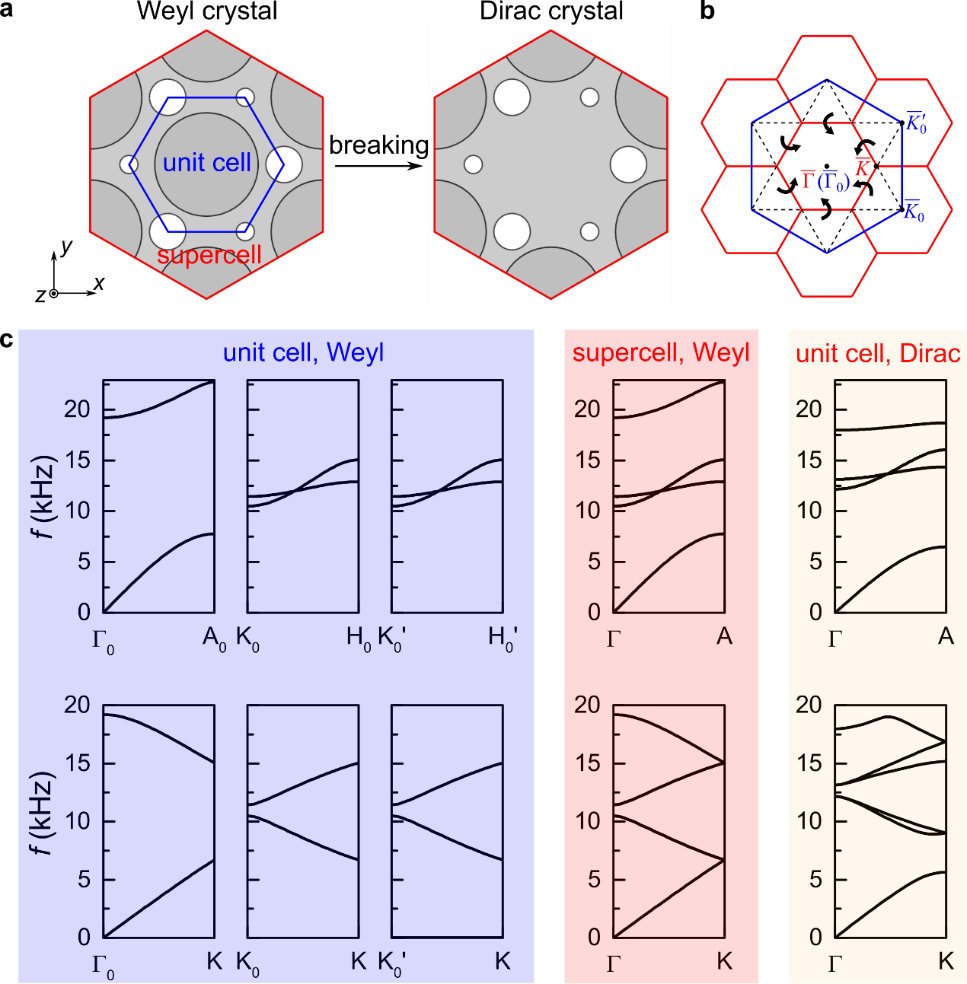


**Fig. S1** **a** Schematic of the original unit cell (blue) and supercell (red) of Weyl acoustic crystal, and the unit cell of 3D Dirac crystal. **b** The Brillouin zone of unit cell (blue) folded into the Brillouin zone of supercell (red). **c** The band dispersion corresponding to the unit cell and supercell of Weyl crystal, and the unit cell of Dirac crystal.

**
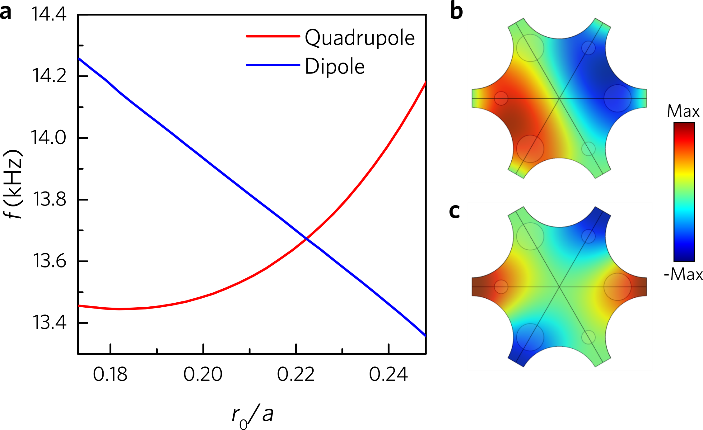
**

**Fig. S2** **a** The relation between the eigenfrequency of dipole/quadrupole modes and $r_{0}/a$ at $\mathbf{k}=(0,0,0.47\pi/h)$. **b,c** Pressure field distribution of **b** dipole and **c** quadrupole mode.


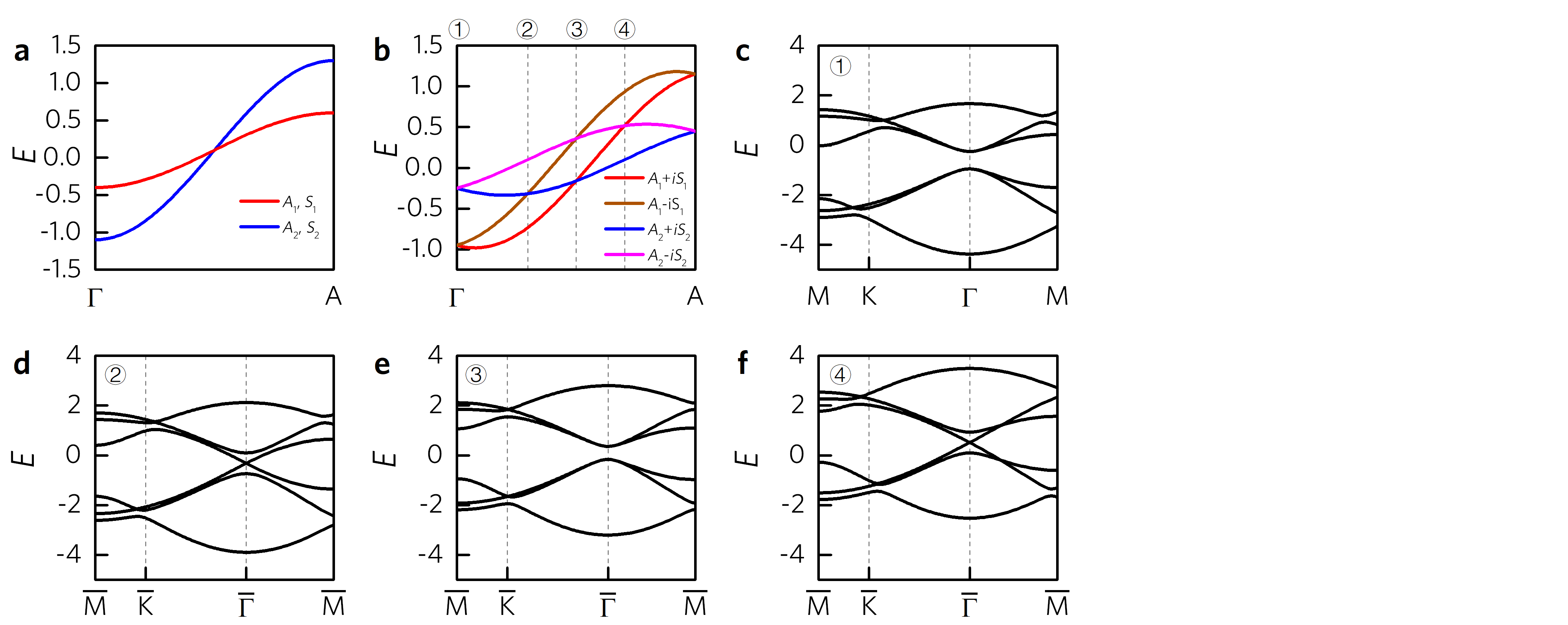


**Fig. S3** Tight-binding calculation of band dispersion. **a** Dispersion for Dirac and **b** Weyl sonic crystals along $\Gamma A$. **c**-**f** Dispersion for Weyl sonic crystals at **c** $k_{z}=0$, **d** $0.297\pi/h$, **e** $0.5\pi/h$ and **f** $0.703\pi/h$, where the bands cross at $\bar{\Gamma}$($\Gamma A$).


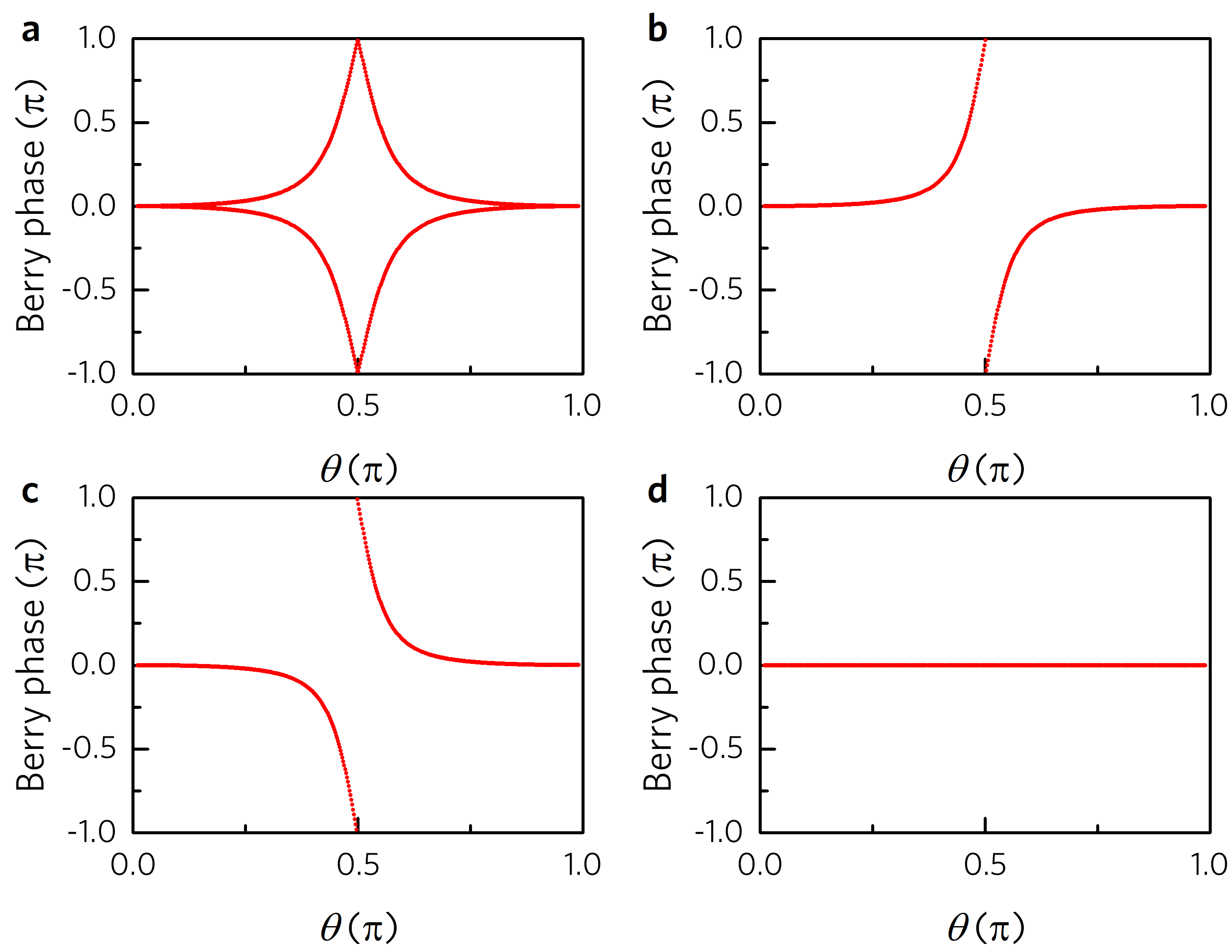


**Fig. S4** Berry phase over a sphere enclosing the crossing point, as a function of polar angle *θ*. **a** Berry phase of the two lower bands around Dirac point (0, 0, 0.5*π*/*h*). **b**,**c** Berry phase of the lower band around **b** WP1 and **c** WP2. **d** Berry phase of the lower band around the crossing between $A_{1}+iS_{1}$ and $A_{2}+iS_{2}$ in Weyl crystal. The radius of the sphere is 0.05*π*/*h*.


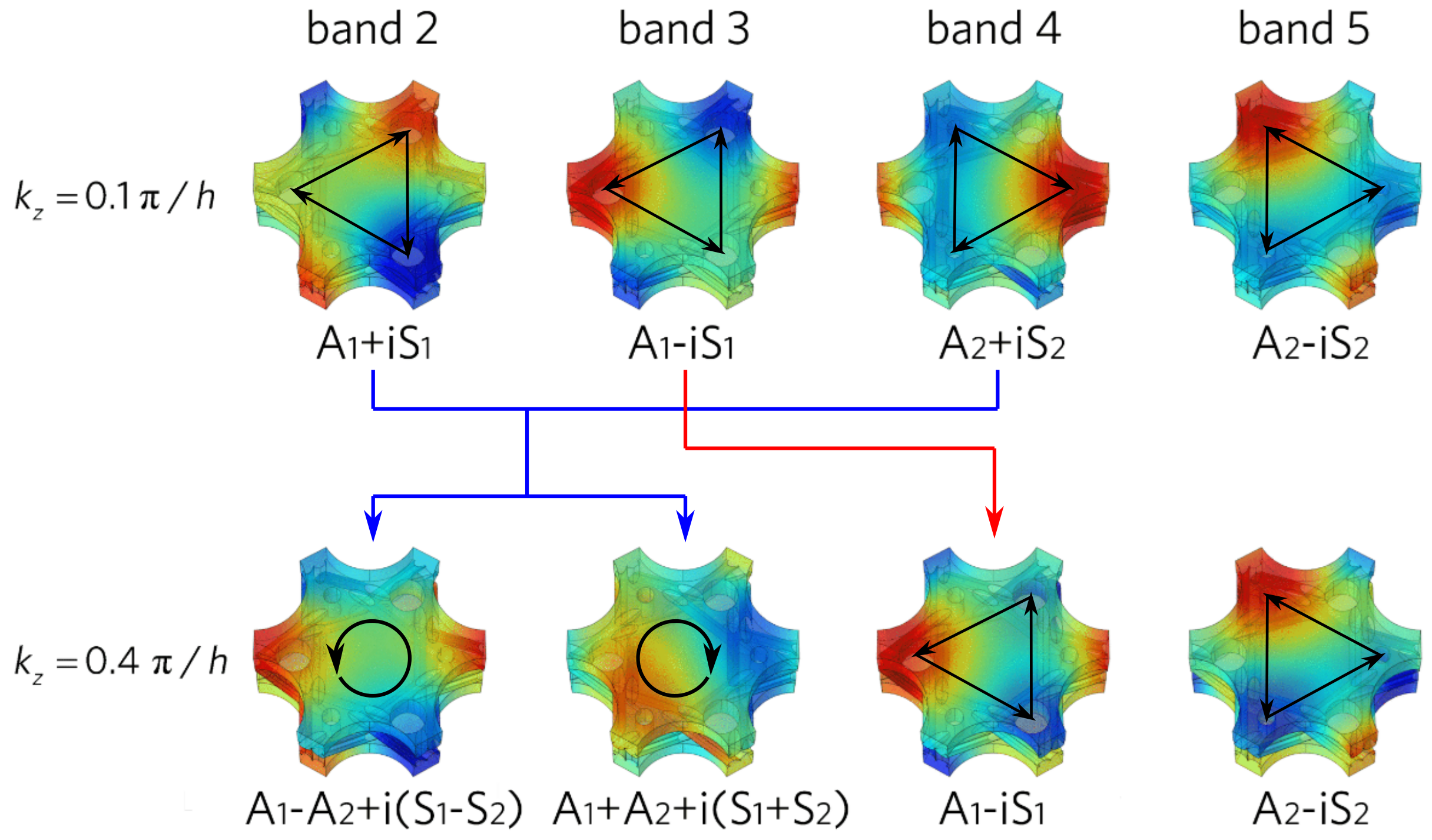


**Fig. S5** Band inversion after $k_{z}$ crosses WP1. $A_{1}-iS_{1}$ moves to the higher band when $A_{2}+iS_{2}$ moves to the lower band and hybridizes with $A_{1}+iS_{1}$.

See Supplemental Movie 1


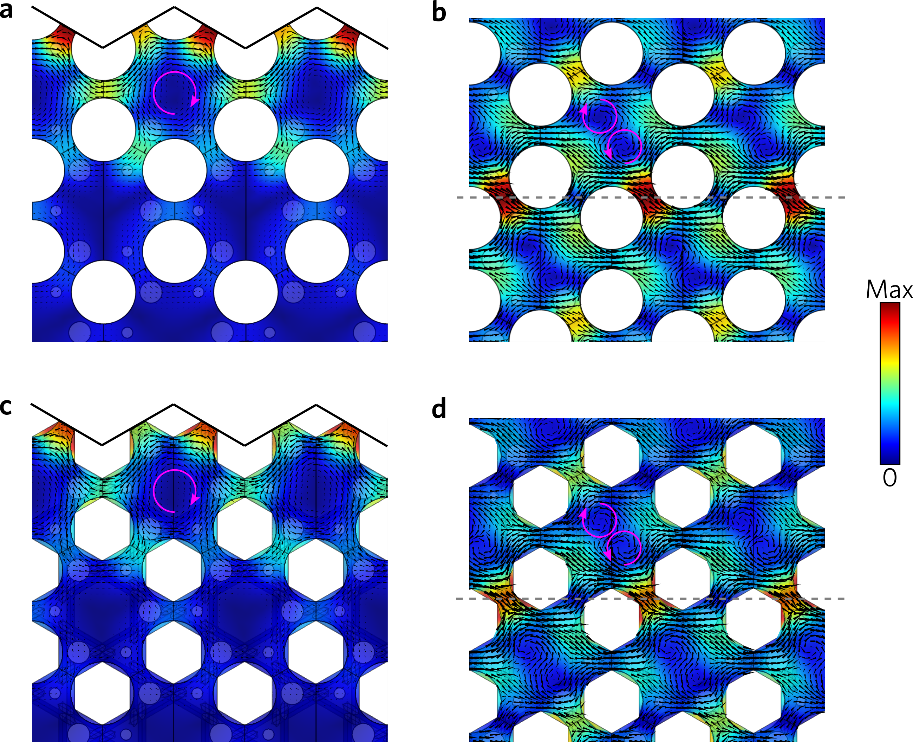


**Fig. S6** Pressure amplitude distribution (colour) and in-plane time-averaged Poynting vectors (black arrows) for **a**,**b** Dirac and **c**,**d** Weyl sonic crystal with **a**,**c** zigzag hard boundary and **b**,**d** zigzag interface. The surface states are traveling rightwards with wave vector $(k_{x},k_{z})=(0.3\pi/a,0)$.

**
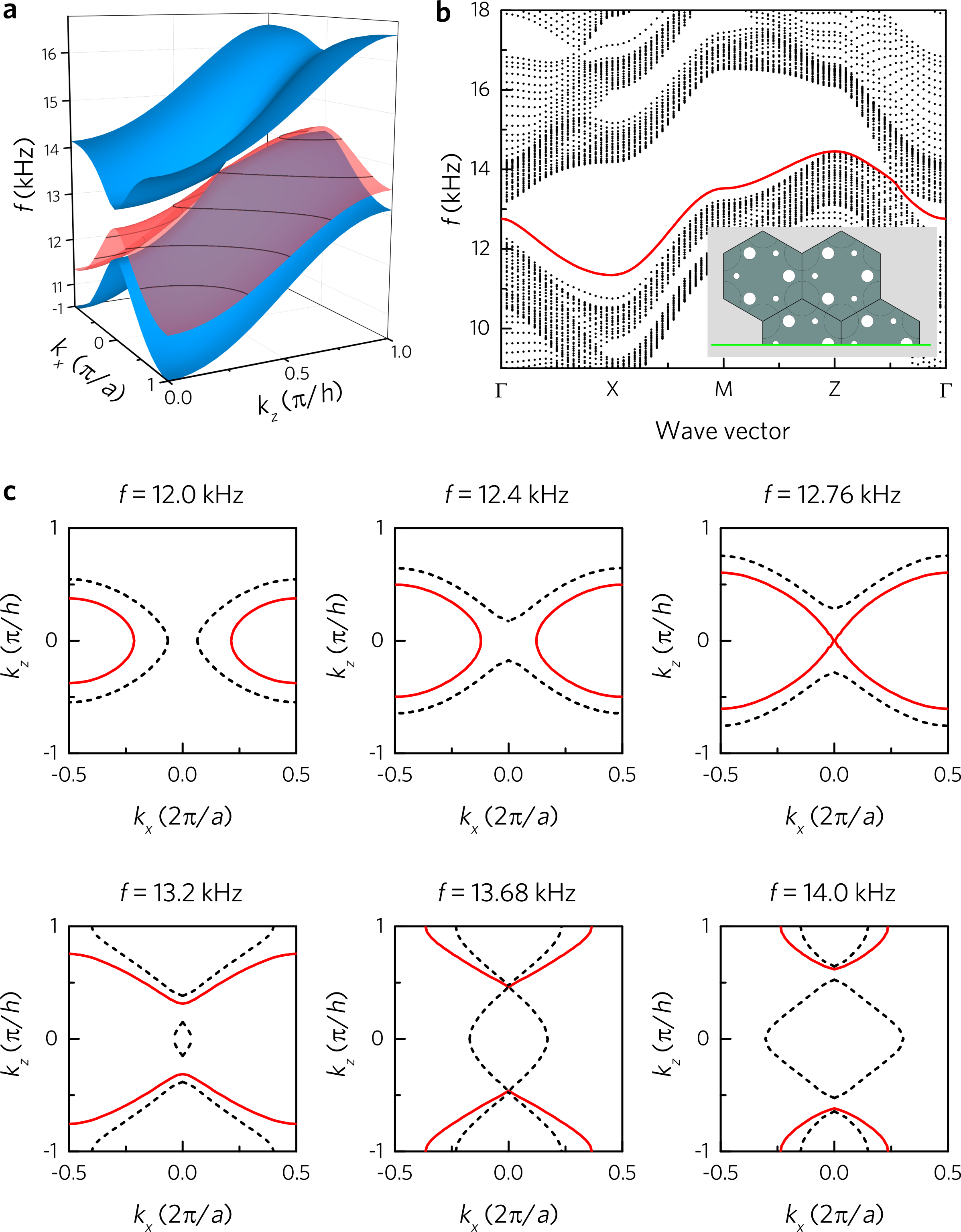
**

**Fig. S7** Surface bands of the 3D Dirac sonic crystal with flat boundary. **a** 3D surface band structure of projected bulk states (blue) and surface states (red). **b** Surface band structure along high symmetry line of surface BZ. Inset shows the schematic of flat boundary. **c**-**e** Equifrequency contours of surface bands among various frequencies. The red lines denote the surface band, and the dashed black lines denote the outline of projected bulk bands.


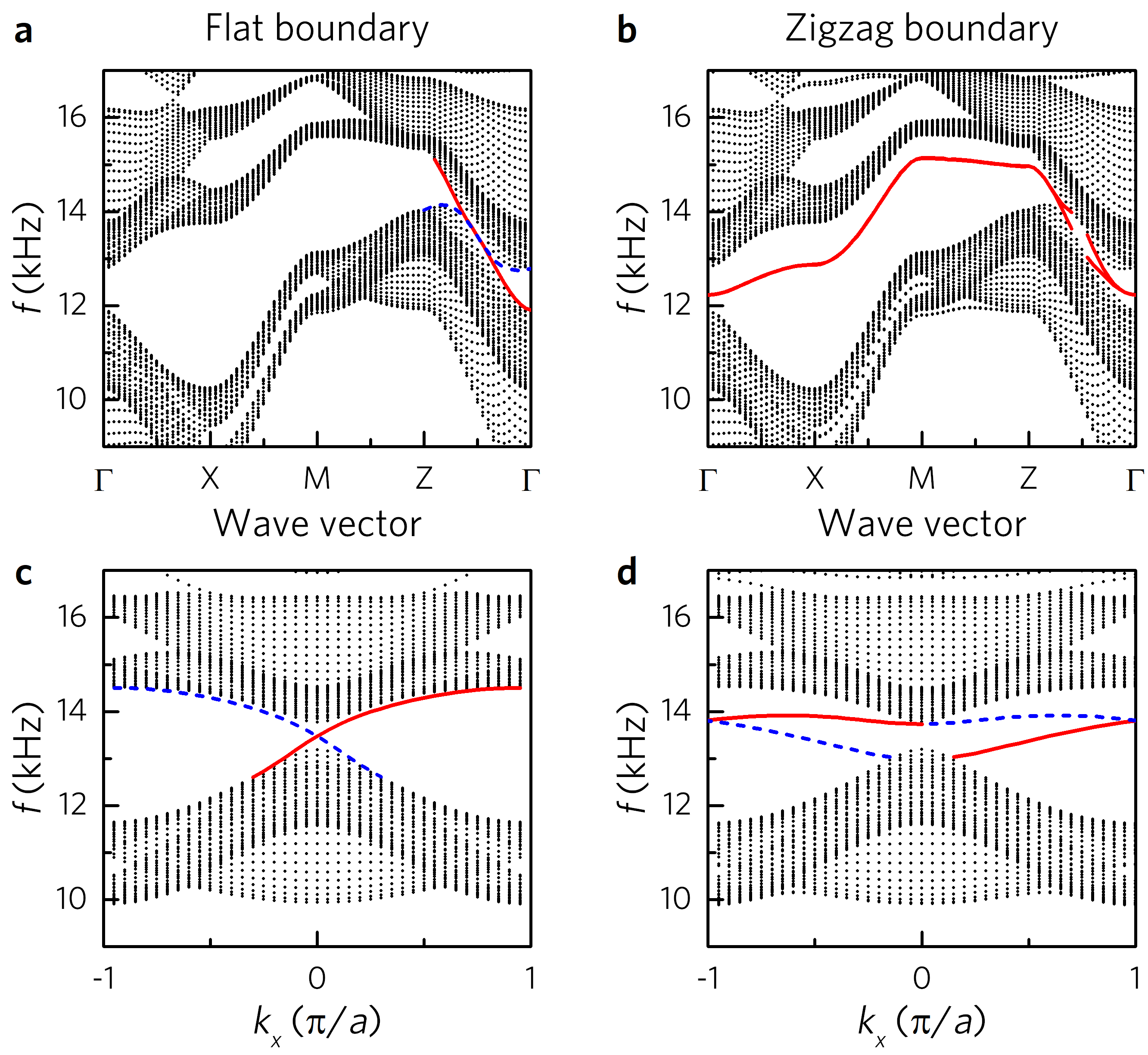


**Fig. S8** Surface bands of the Weyl sonic crystal. **a**,**b** Band dispersion along high symmetry line of surface BZ. **c**,**d** Band dispersion at $k_{z}=0.5\pi/h$. **a**,**c** are with flat boundary, while **b**,**d** are with zigzag boundary. The red lines and blue dashed lines are the surface states, in which the blue dashed lines denote the surface states on the opposite surface.


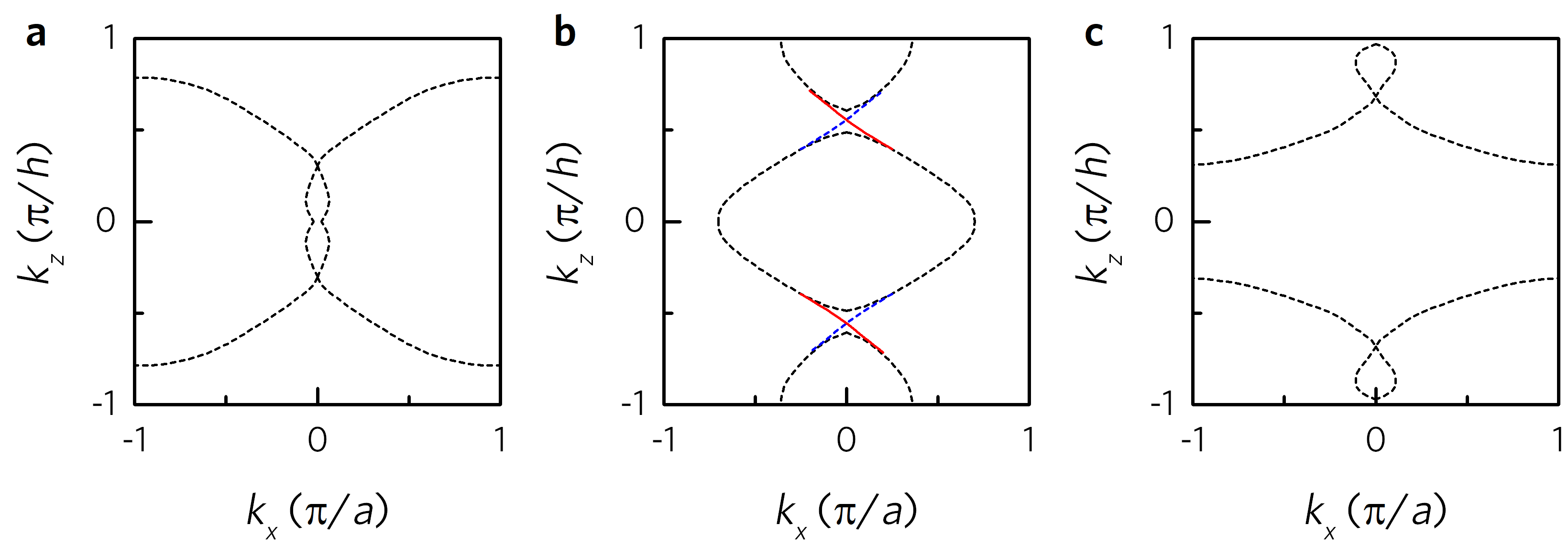


**Fig. S9** Equifrequency contours of the surface states of flat boundary at frequencies of **a** 12.76 kHz (WP1), **b** 13.68 kHz, and **c** 14.06 kHz (WP2).


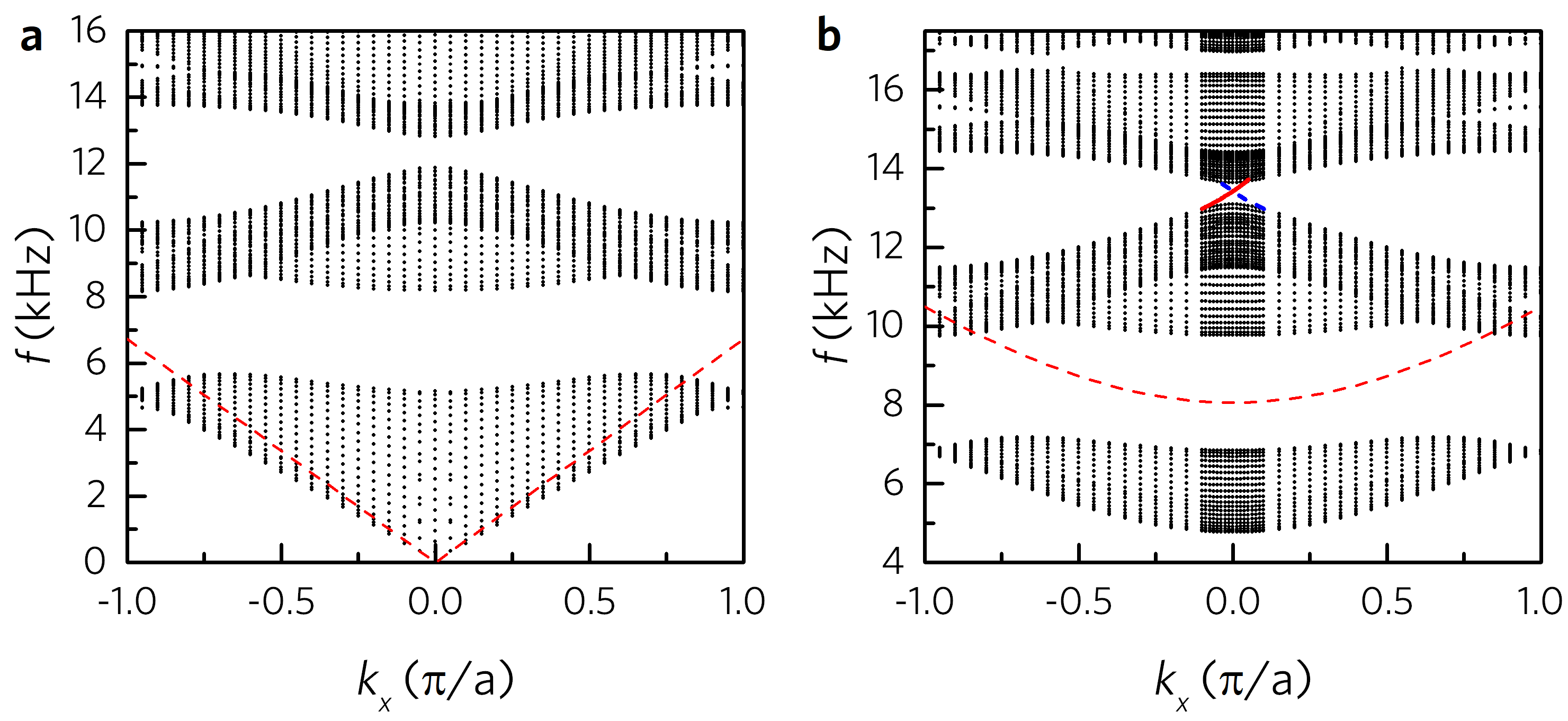


**Fig. S10** Surface state dispersion of Weyl sonic crystal with zigzag open boundary for **a** $k_{z}=0$ and **b** $k_{z}=0.47\pi/h$. The red dashed lines denote the acoustic cone of the airborne sound. The surface modes above the acoustic cone are leaky.


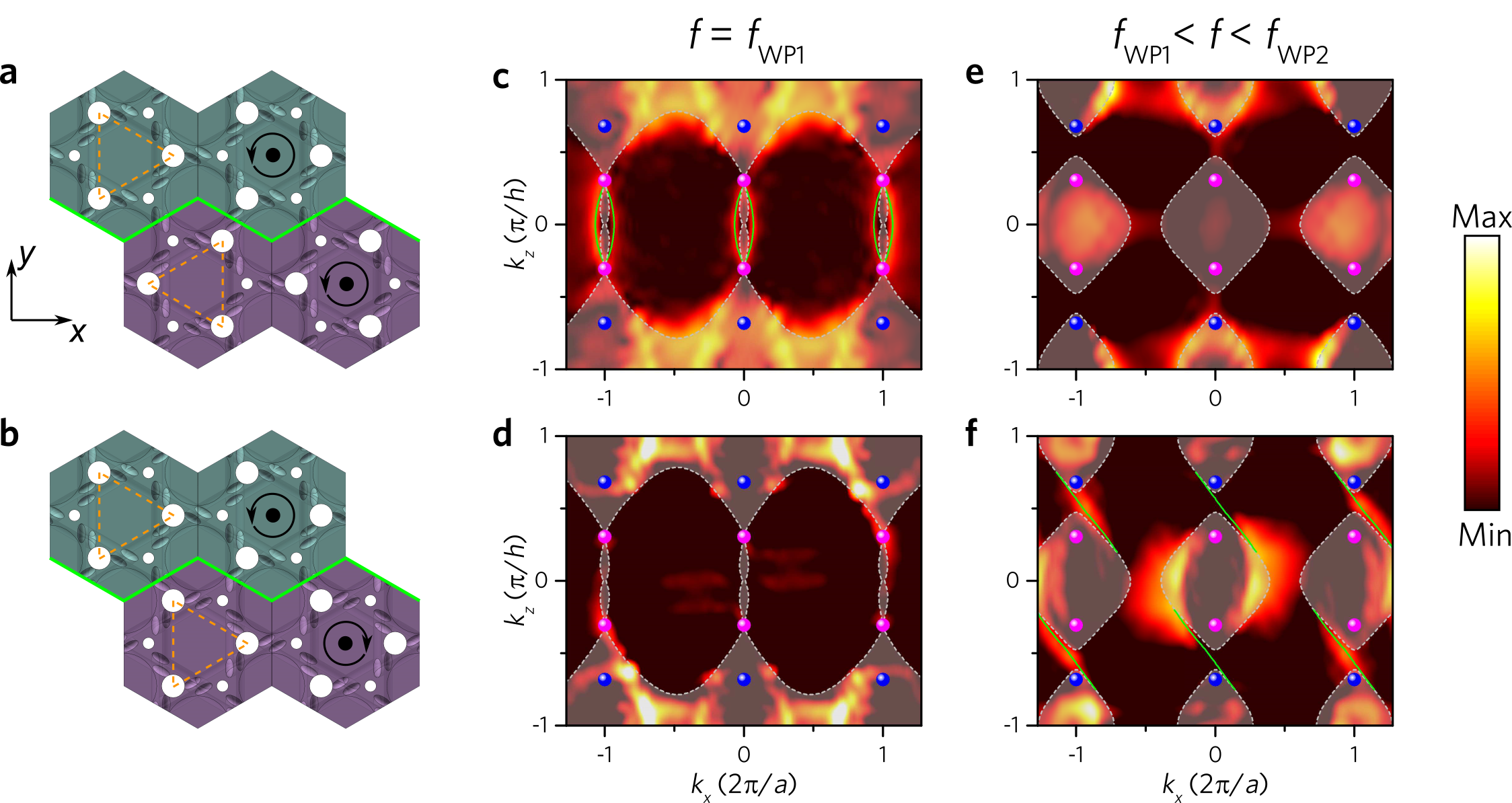


**Fig. S11** **a**,**b** Interface created by inversing only **a** the pseudospin or **b** the chirality. **c**,**d** Equifrequency contours of interface states at 12.76 kHz (WP1), and **e**,**f** at 13.68 kHz between the Weyl frequencies.
