# Supplementary figures and images for: Dirac points and the transition towards Weyl points in three-dimensional sonic crystals
Source: Light Sci Appl. 2020 Dec 22;9:201. doi: 10.1038/s41377-020-00416-2 (PMC7755923; doi:10.1038/s41377-020-00416-2)

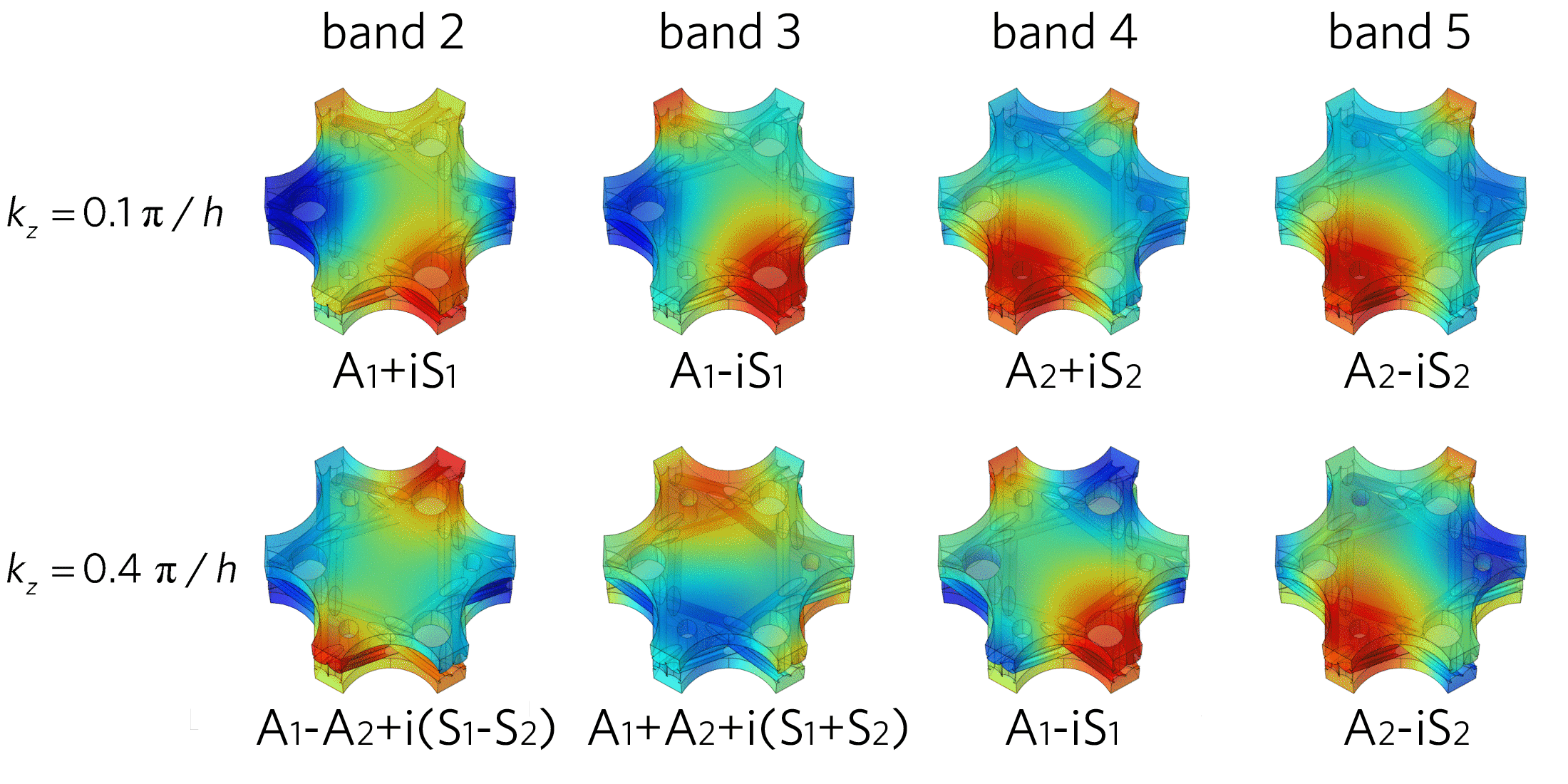

Supplement: Supplementary file 2 — Supplementary Movie 1 [file 41377_2020_416_MOESM2_ESM.gif]
